# Supplementary material for: Molecular and Metabolic Mechanism of Low-Intensity Pulsed Ultrasound Improving Muscle Atrophy in Hindlimb Unloading Rats
Source: Int J Mol Sci. 2021 Nov 9;22(22):12112. doi: 10.3390/ijms222212112 (PMC8625684; doi:10.3390/ijms222212112)

Supplementary Material

Supplementary Figures of RT-PCR

(A) Melt curve and amplification curve of MSTN

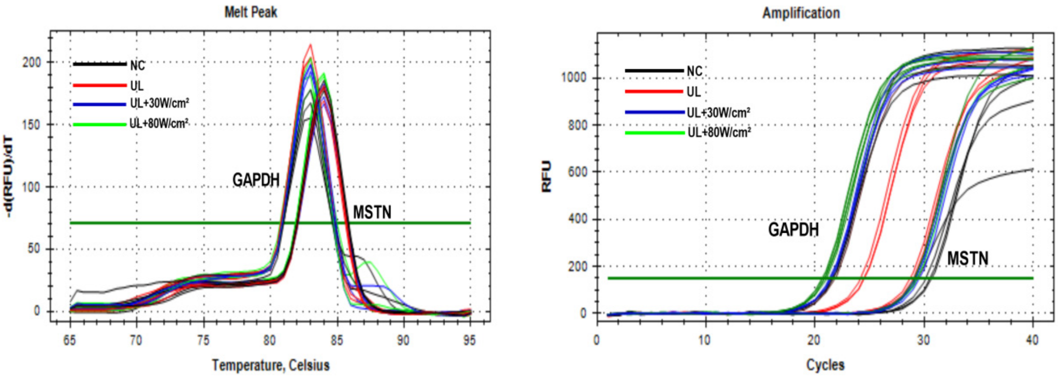

(B) Melt curve and amplification curve of ActrⅡB

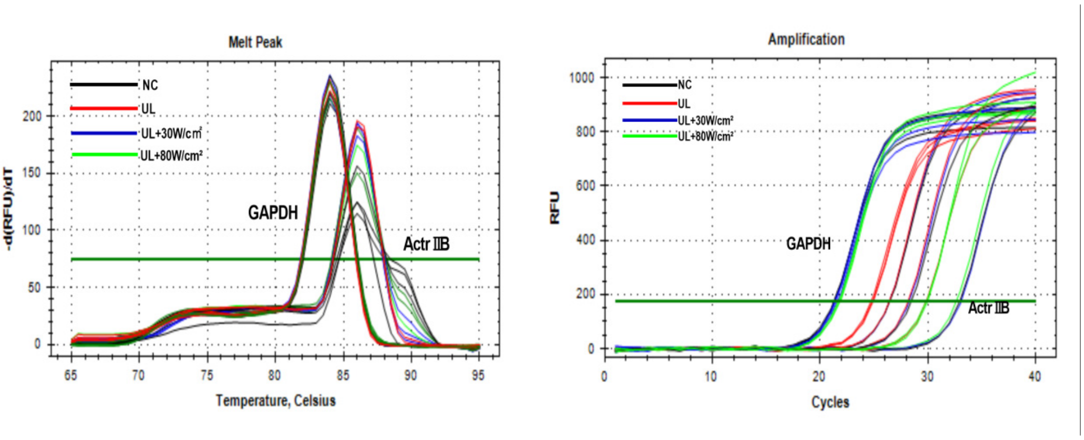

Supplementary Figures of Western blot

Fig 5(C) MyHC3

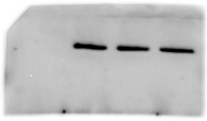

GAPDH

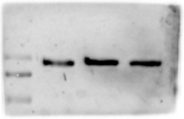

Fig.6(C) MSTN

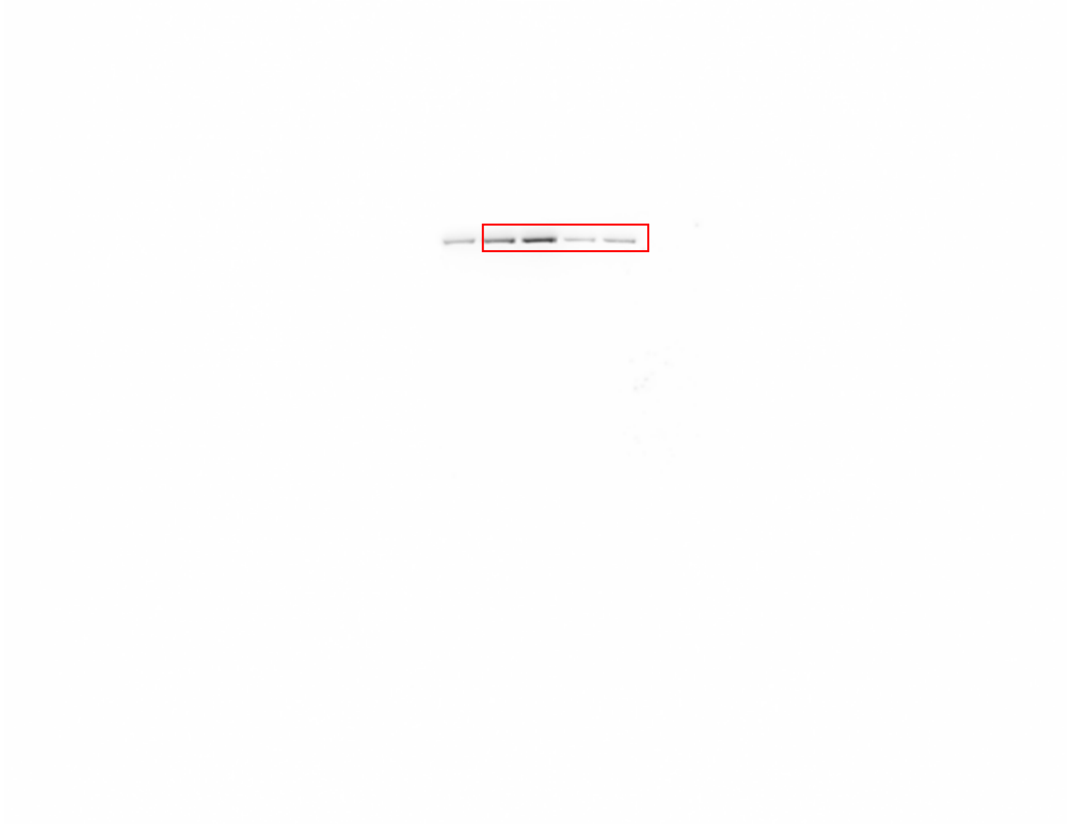

GAPDH

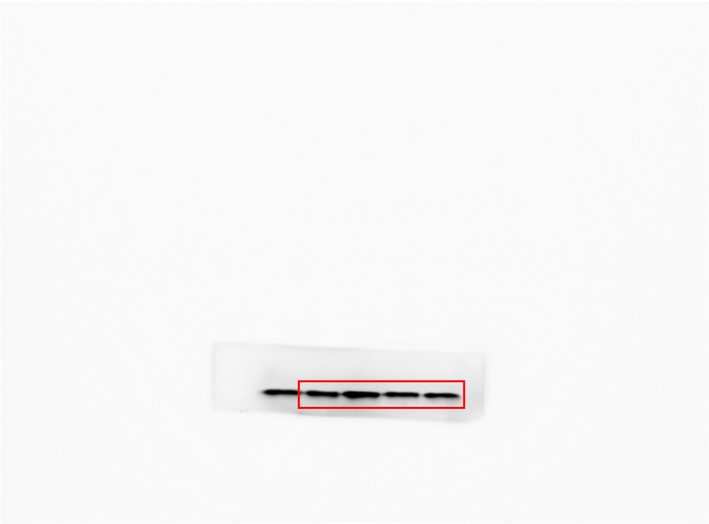

Fig.6（D）ActrⅡB

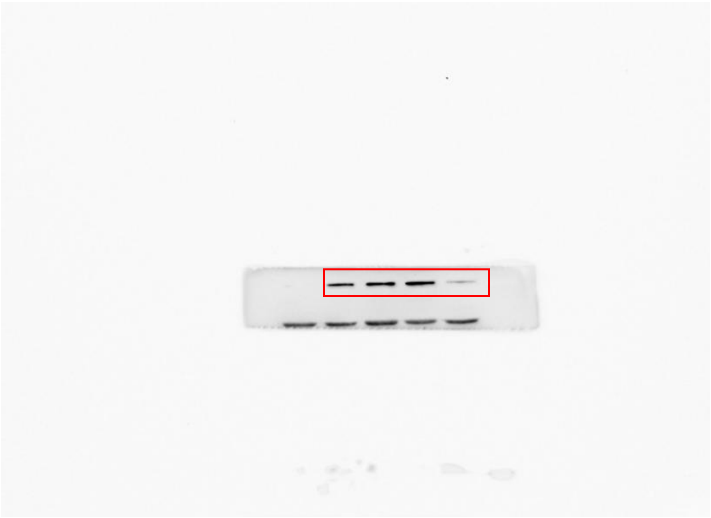

GAPDH

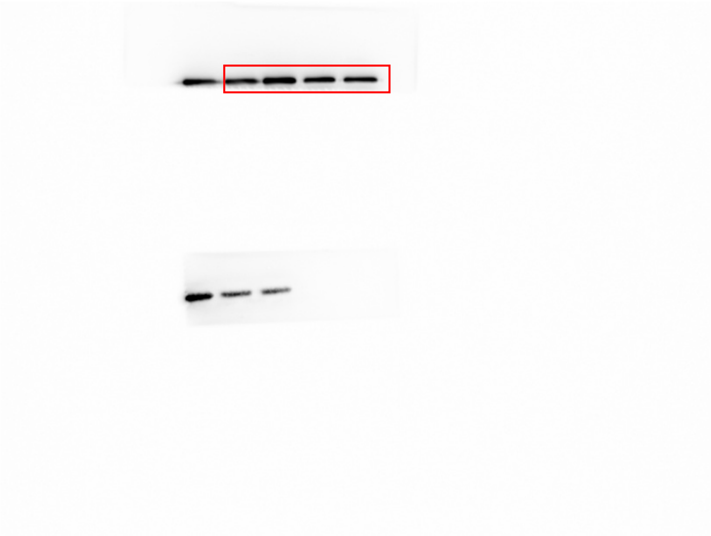

Fig.6（E）AKT

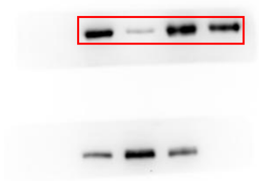

GAPDH

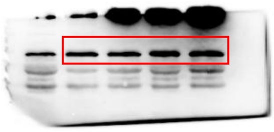

Fig.6（F）mTOR

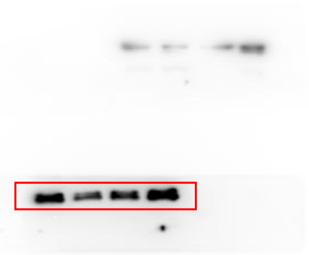

GAPDH

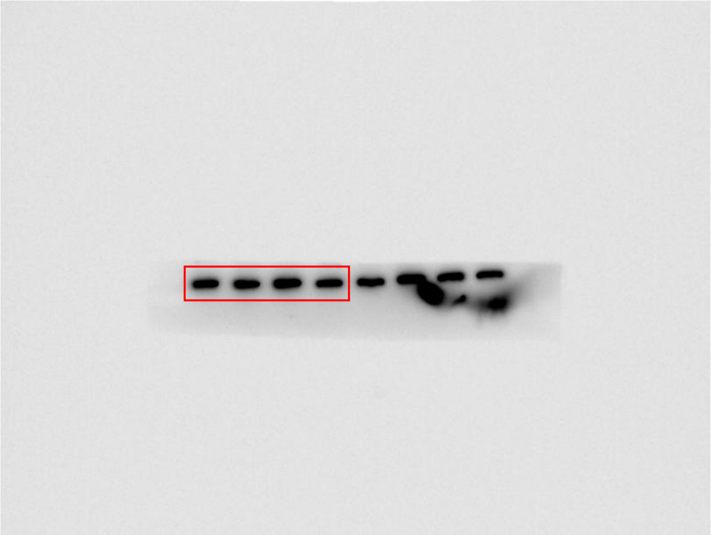

Supplement: Supplementary file 1 [file ijms-22-12112-s001.zip › ijms-1406567-supplementary.pdf]
